# Trends in incidence and mortality of laryngeal cancer in china from 2004 to 2018: Projections to 2033 and decomposition analysis

**DOI:** 10.1371/journal.pone.0318423

**Published:** 2025-02-14

**Authors:** Ze-Xing Zhang, Chun-Ming Huang, Ya-Xi Suo, Long Xie

**Affiliations:** 1 State Key Laboratory of Oral & Maxillofacial Reconstruction and Regeneration, Key Laboratory of Oral Biomedicine Ministry of Education, Hubei Key Laboratory of Stomatology, School & Hospital of Stomatology, Wuhan University, Wuhan, Hubei, China; 2 Stomatology Center, Tongji Hospital, Tongji Medical College, Huazhong University of Science and Technology, Wuhan, Hubei, China; National center for chronic and non-communicable diesease prevention and control, CHINA

## Abstract

This study analyzed trends in the incidence and mortality of laryngeal cancer (LC) in China from 2004 to 2018, provided projections up to 2033, and identified contributing factors influencing these trends. LC data were obtained from the Chinese Cancer Registry Annual Reports for 2004 to 2018. Estimated Annual Percent Change (EAPC) and 95% confidence intervals (CI) were calculated using Joinpoint Regression Software to evaluate trends. LC incidence and mortality rates in registry areas, combined with national population data, were used to estimate new cases and deaths. Projections were made to 2033, and a decomposition analysis identified factors influencing trends. From 2004 to 2018, the age-standardized incidence rate (ASIR) of LC decreased from 1.29 to 1.14 per 100,000, with an EAPC of −1.75% (95% CI: −2.36% to −1.12%). The age-standardized mortality rate (ASMR) also declined from 0.67 to 0.61 per 100,000, with an EAPC of −0.76% (95% CI: −1.28% to −0.25%). Mortality among males aged 60–69, however, showed a significant increase. Population growth and aging contributed to the rise in LC cases and deaths, while epidemiological factors partially offset these increases. By 2033, ASIR and ASMR for males and ASIR for females are projected to decrease, while ASMR for females is expected to rise. ASIR and ASMR in China showed a declining trend from 2004 to 2018, with further decreases projected through 2033. Yet, rising mortality in older males underscores the need for targeted prevention and early detection. Population aging and growth are key drivers of the LC burden, although epidemiological improvements have helped mitigate case increases. Comprehensive public health strategies remain essential to reduce LC impact in China.

## Introduction

In 2019, laryngeal cancer (LC) accounted for 209,149 new cases and 123,356 deaths globally, with an age-standardized incidence rate (ASIR) of 2.5/100,000 and an age-standardized mortality rate (ASMR) of 1.5/100,000 [1]. LC remains a significant public health challenge globally and within China. As a major component of head and neck malignancies, LC accounts for 1–5% of all malignant tumors [2]. The incidence rates of LC have shown notable geographic variation, reflecting differences in risk factor exposure and access to medical resources [1]. Although some regions experienced declines in LC incidence, largely attributed to reduced smoking rates, the burden remains particularly high in countries with less stringent tobacco control policies and among populations with specific genetic and environmental vulnerabilities [3]. In China, LC presented a critical issue due to the country’s large population and significant disparities in healthcare access and lifestyle behaviors across regions[4].

The risk factors for LC have been well established, encompassing both environmental and biological determinants. Tobacco smoking is the most influential risk factor, with alcohol consumption also playing a crucial role, particularly when combined with smoking [5]. Additional environmental factors such as occupational exposure to hazardous substances (such as asbestos and exposure to air pollution) have also been linked to LC [6,7]. Moreover, the role of human papillomavirus (HPV) in the etiology of LC is becoming increasingly recognized, adding complexity to prevention efforts [8]. These risk factors collectively influence incidence of LC, but their relative impact varies across populations due to differences in lifestyle, genetic susceptibility, and exposure levels.

Epidemiological studies on LC have been extensively conducted abroad, providing valuable information that guides clinical work as well as prevention and treatment efforts [9,10]. However, comprehensive surveys on the incidence and mortality rates of LC across the entire Chinese population remain limited, especially in the context of population aging. This study aims to analyze trends in LC incidence and mortality in China from 2004 to 2018, with projections extending to 2033. A decomposition analysis was conducted to identify the primary factors contributing to these trends, providing a scientific basis for policy-making and resource allocation to address the ongoing challenge of LC in China’s aging population.

## Methods

### Data collection

Data on LC (ICD-10 code C32) were obtained from the Chinese Cancer Registry Annual Reports covering the period from 2004 to 2018. This dataset provided detailed counts of new LC cases, deaths, and the age-standardized rates (ASR) for both incidence and mortality, categorized by gender and age group. Age-standardization was conducted using the world standard population (Segi’s population) to allow for temporal and regional comparisons.

The data collection follows strict quality control protocols to ensure accuracy and completeness. The registry complies with international cancer registration standards and follows the guidelines set by the International Agency for Research on Cancer (IARC) [11]. Population estimates for China were derived from Global Health Data Exchange [12]. Ethics approval and participant consent were not applicable for this study as the data used were obtained from a publicly available database.

### Statistical analysis

The trends in the incidence and mortality rates of LC across various age groups were assessed using the Estimated Annual Percent Change (EAPC) and the corresponding 95% confidence intervals (CI), as well as the age-standardized incidence rate (ASIR) and age-standardized mortality rate (ASMR). The EAPC was calculated using Joinpoint Regression Software (version 4.9.0.0), which identifies significant changes in trends and quantifies the annual rate of change during the study period from 2004 to 2018 [13]. EAPC values could not be computed for incidence rates among males under 30 years and females under 40 years, as well as for mortality rates among males under 30 years and females under 50 years, due to the presence of zero values in the dataset.

To project the ASIR and ASMR of LC up to 2033, Bayesian age-period-cohort (BAPC) models were employed. This method allows for the inclusion of temporal patterns (age, period, and cohort effects) and generates probabilistic projections based on observed trends. The BAPC models were fitted using the Integrated Nested Laplace Approximation (INLA) method in R software (version 4.1.3), providing reliable estimates for future trends in LC incidence and mortality in China [14].

A decomposition analysis was conducted to assess the factors contributing to changes in LC cases and deaths from 2004 to 2018. The analysis disaggregated the effects of population growth, population aging, and changes in incidence and mortality rates [15]. This allowed for quantification of how demographic changes and epidemiological factors influenced the overall burden of LC.

All data were analyzed using software R. A difference was considered significant when the 95% CI excluded zero, and statistical significance was set at p <  0.05.

## Results

### Cases and trends of LC from 2004 to 2018 in cancer registries

**Fig 1:**
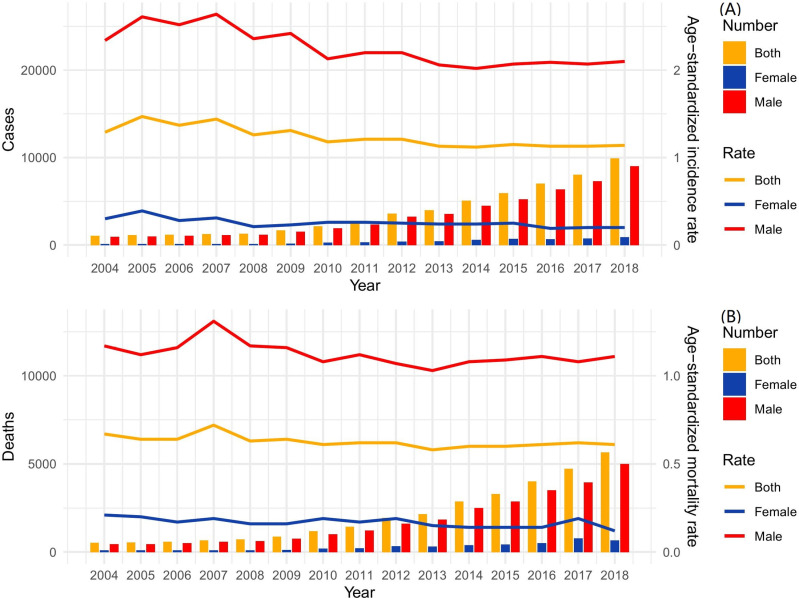
Cases and deaths of LC from the Chinese Annual Cancer Registry, 2004–2018. (A) Incidence; (B) Death.

From 2004 to 2018, the number of new LC cases in the cancer-registered regions of China increased from 1,045 to 9,898, representing an 8.47-fold increase (Fig 1A). However, the ASIR decreased from 1.81 to 1.14 per 100,000, representing a 37.02% decline and an EAPC of −1.75% (95% CI: −2.36% to −1.12%) (Table 1).

**Table 1:** Burden and trends of LC in the chinese annual cancer registry in 2004 and 2018.

		2004		2018		Changes		
Measure	Gender	Number	ASR per 100,000	Number	ASR per 100,000	Change in absolute number (times)	Change in absolute rate (%)	2004–2018 EAPC of ASR (95%CI)
Incidence	Both	1,045	1.81	9,898	1.14	8.47	−37.02	−1.75(−2.36 to −1.12)
	Male	913	2.34	8,994	2.10	8.85	−10.26	−1.70(−2.35 to −1.05)
	Female	132	0.30	904	0.20	5.85	−33.33	−3.23(−4.79 to −1.64)
Death	Both	526	0.99	5652	0.61	9.75	−38.38	−0.76(−1.28 to −0.25)
	Male	437	1.17	4993	1.11	10.43	−5.13	−0.72(−1.34 to −0.10)
	Female	89	0.21	659	0.12	6.40	−42.86	−2.45(−3.99 to −0.89)

Among males in the registry regions, the new cases of LC increased from 913 to 8,994, representing an 8.85-fold rise (Fig 1A), while the ASIR decreased from 2.34 to 2.10 per 100,000, indicating a 10.26% reduction and a EAPC of −1.70% (95% CI: −2.35% to −1.05%) (Table 1).

In females, new cases of LC increased from 132 to 904, representing a 5.85-fold increase (Fig 1A). Concurrently, the ASIR declined from 0.30 to 0.20 per 100,000, indicating a 33.33% reduction with an EAPC of −3.23% (95% CI: −4.79% to −1.64%) (Table 1).

### Deaths, and trends of LC from 2004 to 2018 in cancer registries

In the registered regions of China, the number of deaths from LC increased from 526 in 2004 to 5,652 in 2018, representing a 9.75-fold increase (Fig 1B). However, the ASMR declined from 0.99 to 0.61 per 100,000, indicating a 38.38% reduction and a downward trend. This decrease was characterized by an EAPC of −0.76% (95% CI: −1.28% to −0.25%) (Table 1).

The number of male deaths from LC increased from 437 in 2004 to 4,993 in 2018, a 10.43-fold increase (Fig 1B), but the ASMR decreased from 1.17 to 1.11 per 100,000, a 5.13% reduction, showing a downward trend, with an EAPC of −0.72% (95% CI: −1.34% to −0.10%) (Table 1).

In females, the number of deaths from LC increased from 89 in 2004 to 659 in 2018, a 6.40-fold increase (Fig 1B). However, the ASMR decreased from 0.21 to 0.12 per 100,000, a 42.86% decrease, showing a downward trend, with an EAPC of −2.45% (95% CI: −3.99% to −0.89%) (Table 1).

### Incidence and mortality by age group in cancer registries

**Fig 2:**
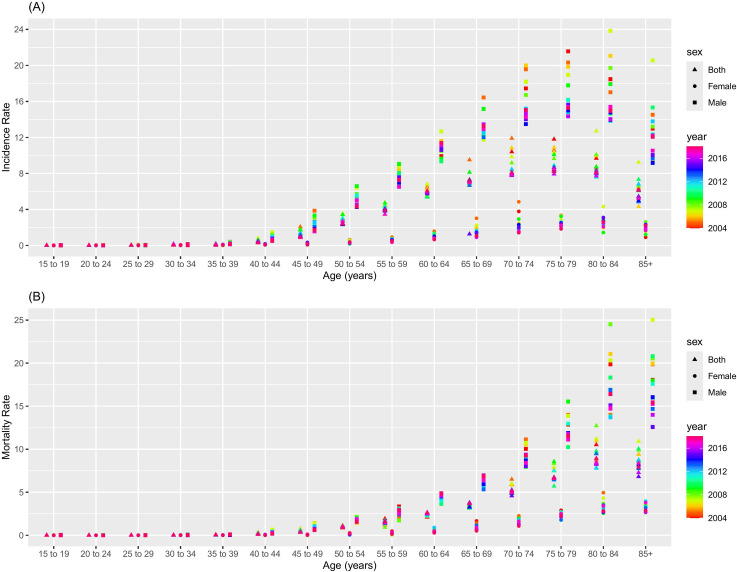
The incidence and mortality data of LC by age group from the Chinese Annual Cancer Registry, 2004–2018. (A) Incidence; (B) Mortality.

From 2004 to 2018, the incidence and mortality rates of LC were consistently higher in males than in females across all age groups. Generally, the incidence of LC initiated in the 40–44 age bracket and increased progressively with advancing age (Fig 2A). Similarly, mortality rates began to rise in the 45–49 age group and increased with age (Fig 2B).

### The incidence and mortality trend analysis of LC by age group in cancer registries

**Fig 3:**
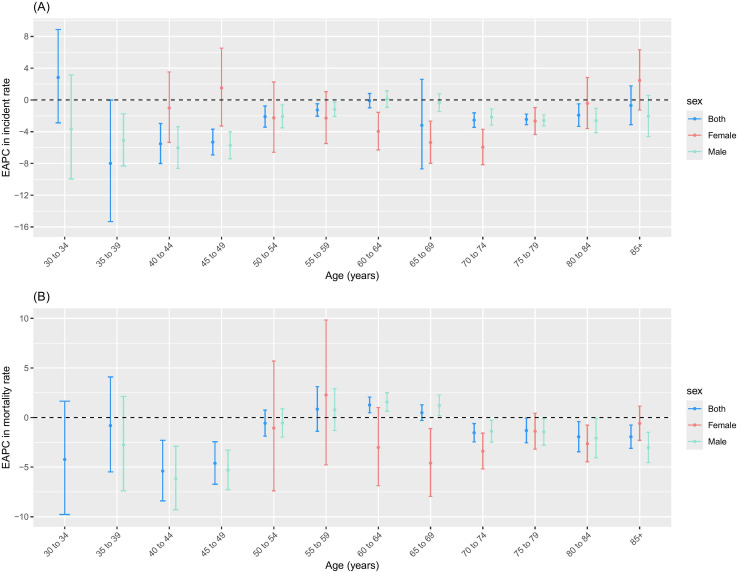
EAPC in incidence and mortality of LC for age group. (A) Incidence; (B) Mortality.

The incidence rates of LC remained stable or demonstrated a downward trend across all age groups above 15 years. The most significant decline was observed in the 45–49 age group for both sexes, with an EAPC of −5.32% (95%CI: −6.92% to −3.69%). Among them, the fastest decrease was noted in males aged 45 to 49, with an EAPC of −5.73% (95%CI: −7.41% to −4.02%), and in females aged 70 to 74, with an EAPC of −5.36% (95%CI: −7.99% to −2.67%) (Fig 3A).

The mortality rates for LC largely remained stable or showed a declining trend across age groups above 15 years. The most pronounced decrease was observed in the 40–44 age group for both sexes, with an EAPC of −5.39% (95%CI: −8.40% to −2.30%). However, a significant upward trend was noted in the 60–64 age group, with an EAPC of 1.27% (95%CI: 0.48% to 2.06%). Among them, the fastest decline was seen in males aged 40 to 44, with an EAPC of −6.15% (95%CI: −9.30% to −2.90%), and in females aged 65 to 69, with an EAPC of −4.59% (95%CI: −7.96% to −1.11%). On the other hand, an increasing trend in mortality was observed in the 60–64 and 65–69 age groups among males, with EAPC values of 1.27% (95%CI: 0.48% to 2.06%) and 1.22% (95%CI: 0.18% to 2.27%), respectively (Fig 3B).

### Decomposition of changes in LC cases and deaths, 2004 to 2018

Table 2 presents the results of a decomposition analysis, examining the changes in the number of incident cases and deaths from LC and the proportions of these changes attributable to three population-level determinants: population aging, population growth, and epidemiological changes.

**Table 2:** Changes in LC cases and deaths in male and female from 2004 to 2018, decomposed by three population-level determinants: population aging, population growth and epidemiological changes.

Metric	sex	Overall difference	Aging (Percent %)	Population (Percent %)	Epidemiological change (Percent %)
LC cases	Both	7118.74	8296.86 (116.55)	2093.64 (29.41)	−3271.76 (−45.96)
	Male	6749.24	7326.71 (108.56)	1807.04 (26.77)	−2384.51 (−35.33)
	Female	68.27	917.05 (1343.28)	229.05 (335.50)	−1077.84 (−1578.78)
LC deaths	Both	4518.77	4771.22 (105.59)	1125.91 (24.92)	−1378.36 (−30.50)
	Male	4249.29	4042.50 (95.13)	927.74 (21.83)	−720.95 (−16.97)
	Female	33.86	719.93 (2126.21)	161.40 (476.67)	−847.48 (−2502.89)

**Fig 4:**
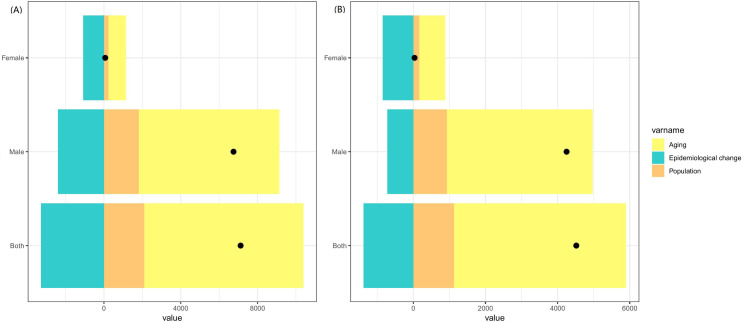
Contribution of changes in population aging, population growth, and age-speciﬁc incidence rate to changes in incident cases of LC from 2004 to 2018 in China. (The decomposition was conducted using the number of cases and deaths in 2004 as the reference for each year) (A) Incidence; (B) Death.

Between 2004 and 2018, 116.55% of the increase in the number of LC cases was attributed to population aging, followed by population growth (29.41%). Conversely, epidemiological changes (i.e., decreases in age-specific incidence rates for most age groups) resulted in a reduction of 45.96% in the population cases (Fig 4A).

Between 2004 and 2018, 105.59% of the increase in the number of LC deaths was attributed to population aging, followed by population growth (24.92%). Conversely, epidemiological changes (i.e., decreases in age-specific mortality rates for most age groups) resulted in a reduction of 30.50% in the population deaths (Fig 4B).

### Projection up to 2033

According to projections, the number of new cases of LC among males and females aged 15 and above will increase from 23,622 and 3,009 in 2019 to 27,582 and 5,582 respectively in 2033 (S1 Table and S2). Similarly, the number of deaths from LC among males and females aged 15 and above will rise from 13,601 and 2,835 in 2019 to 22,225 and 7,815 respectively in 2033 (S3 Table and S4).

**Fig 5:**
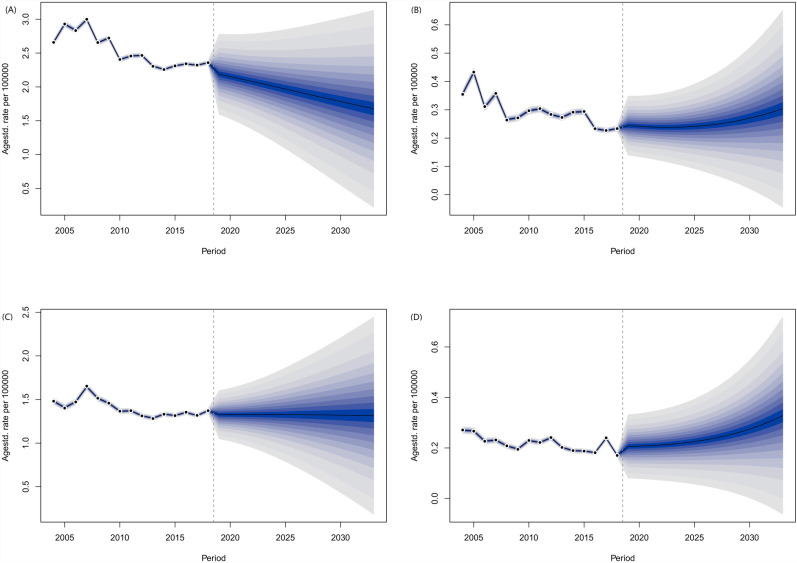
Projected ASIR and ASMR of LC in China over the next 15 years based on the Bayesian APC model. (A) ASIR of male; (B) ASMR of male; (C) ASIR of female; (D) ASMR of female.

The ASIR and ASMR of LC among males aged 15 and above will decrease from 2.19 and 1.33 per 100,000 in 2019 to 1.68 and 1.31 per 100,000 respectively in 2033 (Fig 5A–5B). For females aged 15 and above, the ASIR and ASMR of LC will increase from 0.24 and 0.21 per 100,000 in 2019 to 0.30 and 0.33 per 100,000 respectively in 2033 (Fig 5C–5D).

## Discussion

Our study provides a detailed analysis of LC trends in China, highlighting both increases in absolute numbers of cases and deaths and decreases in ASIR and mortality ASMR from 2004 to 2018 in cancer registry regions. This finding suggests that although public health measures may be effective in reducing the relative risk of LC, population growth and aging are driving an overall increase in the burden of the disease. Notably, our results also indicate a rising trend in LC mortality rates among males aged 60 to 69 during this period, which warrants targeted interventions.

The decline in ASIR and ASMR across the general population, particularly among males, can be attributed to several factors, including improved healthcare access, public health initiatives, environmental factors, and particularly, the implementation of stricter tobacco control measures. Tobacco smoking is one of the risk factors for LC, and the decline in male smoking rates in China may have contributed to the observed reduction in incidence and mortality of LC [16,17]. Since 2002, indoor formaldehyde concentrations in residential buildings, schools, and offices across the country have been steadily declining each year [18]. Additionally, China officially signed the Framework Convention on Tobacco Control in 2005, reflecting the government’s increased emphasis on tobacco control [19]. However, the upward trend in mortality among older males suggests that this population may not have benefited equally from these interventions, potentially due to delayed diagnosis, comorbidities, or less effective treatments for advanced stages of LC [20].

In contrast, the slightly increasing trend in both ASIR and ASMR for females aged 15 and above between 2019 and 2033, as projected by our analysis, raises significant public health concerns. Historically, the lower incidence of LC in females has been linked to lower smoking rates compared to males [21]. However, rising cigarettes consumption per day among women in some regions of China could explain this projected increase in LC incidence [22]. Moreover, other factors, such as environmental exposures and occupational hazards, may play a more significant role in female LC cases than previously recognized [7]. These findings highlight the importance of gender-specific prevention strategies, including anti-smoking campaigns and workplace safety measures, to mitigate the growing LC burden among women.

Our decomposition analysis further supports the role of demographic shifts, specifically population aging, in driving the rising absolute numbers of LC cases and deaths. Between 2004 and 2018, population aging accounted for the largest proportion of the increase in LC cases and deaths, while epidemiological improvements have helped offset some of the disease burden [23]. As China’s population continues to age, the absolute number of LC cases and deaths is expected to increase, even if age-standardized rates continue to decline [21]. This demographic shift underscores the urgent need for healthcare systems to be better equipped to handle the increasing cancer burden, particularly in older populations [24].

Another important finding is the upward trend in LC mortality among males aged 60 to 69. This age group represents a vulnerable demographic that may be experiencing less benefit from public health interventions or advancements in medical treatments. Possible reasons for this could include more aggressive forms of cancer in older males or higher levels of exposure to risk factors such as tobacco, alcohol, and occupational hazards earlier in life [3]. Additionally, older patients may face barriers to timely and adequate treatment, such as financial constraints or geographic inaccessibility to specialized care, which can contribute to higher mortality rates [25]. Future efforts should focus on improving early detection and providing accessible treatment options for older adults.

The projected increase in the incidence and mortality rates of LC among Chinese women can be attributed to several factors. One major contributor is the rising prevalence of smoking among women, particularly in certain regions of China. Historically, smoking rates among women were lower, which kept LC incidence down. However, in recent years, increased tobacco consumption by women has become more evident, contributing to this upward trend in cancer cases [22].

Additionally, women may face heightened exposure to secondhand smoke, environmental pollutants, and occupational hazards, such as exposure to chemicals or dust in certain industries, which could also increase the risk of developing LC [26,27]. Other lifestyle factors, including alcohol consumption and dietary patterns, may further exacerbate this risk in the future [28].

Building on the findings of our study, we recommend several targeted policy interventions to address the observed trends in LC incidence and mortality. First, targeted screening programs for older males, particularly those aged 60 to 69, should be implemented to improve early detection and timely treatment. Screening efforts could focus on high-risk populations, such as long-term smokers, individuals with occupational exposures, or those with a family history of LC. Early detection is critical, as it increases the likelihood of successful treatment and reduces mortality rates [29].

Although the ASIR and ASMR of LC in China are lower than the global average, the sheer size of China’s population means that the number of LC cases and deaths remains very high. It is reported that LC cases and deaths in China and India together account for over 30% of the global total, due to their large population bases, underscoring the importance of policies that address shared demographic challenges in these countries [1]. For instance, China’s experiences with tobacco control, environmental regulation, and improved healthcare access could provide valuable insights for India’s health policymakers, particularly as India’s population has now surpassed China’s. However, given the growing aging population in China, it will be critical for China to continue adapting its healthcare systems to effectively manage the increasing LC burden, while India, in turn, must develop prevention and control policies tailored to its unique demographic context.

This study has several strengths, including the use of robust statistical models, such as the BAPC model, which allows for comprehensive projections based on historical data. Additionally, the use of national cancer registry data provides a reliable and representative picture of LC trends across China. However, there are limitations to consider. The quality of cancer registry data may vary across regions, potentially leading to underreporting or misclassification of cases [30]. To address this, China’s cancer registries should strictly adhere to international cancer registration standards when reporting data. Additionally, establishing more cancer registries would help cover a larger population and provide a more accurate reflection of the national disease burden of LC. Furthermore, our projections are based on current trends and do not account for future policy changes (e.g., “Sanming Healthcare Reform”), healthcare improvements, or emerging risk factors that could alter LC incidence and mortality patterns.

## Conclusion

In conclusion, while the ASIR and ASMR of LC in China generally declined from 2004 to 2018, the absolute number of cases and deaths rose due to population growth and aging. The increasing mortality rate among older males, as well as the projected rise in incidence and mortality of LC among females, highlights the need for targeted prevention strategies. Continued efforts in tobacco control, early detection, and gender-specific interventions are critical to further reduce the burden of LC in China. Policymakers must also focus on addressing the healthcare needs of an aging population to mitigate the future impact of this disease.

## Supporting information

S1 Table Estimated age-specific LC cases in China male from 2019 to 2033 based on Bayesian APC prediction model. (DOCX)

S2 Table Estimated age-specific LC cases in China female from 2019 to 2033 based on Bayesian APC prediction model. (DOCX)

S3 Table Estimated age-specific LC deaths in China male from 2019 to 2033 based on Bayesian APC prediction model. (DOCX)

S4 Table Estimated age-specific LC deaths in China female from 2019 to 2033 based on Bayesian APC prediction model. (DOCX)
