# Supplementary material for: Trends in incidence and mortality of laryngeal cancer in china from 2004 to 2018: Projections to 2033 and decomposition analysis
Source: PLoS One. 2025 Feb 14;20(2):e0318423. doi: 10.1371/journal.pone.0318423 (PMC11828402; doi:10.1371/journal.pone.0318423)
Supplement: S1 Table — (DOCX) [file pone.0318423.s001.docx]

**Table S1** Estimated age-specific LC cases in China male from 2019 to 2033 based on Bayesian APC prediction model

| Year | Number of age-specific LOC cases | | | | | | | | | | | | | | | |
| --- | --- | --- | --- | --- | --- | --- | --- | --- | --- | --- | --- | --- | --- | --- | --- | --- |
|  | 15-19 | 20-24 | 25-29 | 30-34 | 35-39 | 40-44 | 45-49 | 50-54 | 55-59 | 60-64 | 65-69 | 70-74 | 75-79 | 80-84 | 85+ | Total |
| 2019 | 5 | 7 | 15 | 44 | 110 | 343 | 1111 | 2577 | 3243 | 4279 | 4441 | 3387 | 2252 | 1260 | 548 | 23622 |
| 2020 | 5 | 7 | 15 | 45 | 117 | 327 | 1041 | 2482 | 3435 | 4076 | 4652 | 3582 | 2307 | 1271 | 578 | 23940 |
| 2021 | 5 | 7 | 14 | 45 | 123 | 317 | 978 | 2374 | 3589 | 3919 | 4815 | 3784 | 2381 | 1290 | 602 | 24243 |
| 2022 | 5 | 7 | 13 | 45 | 128 | 315 | 925 | 2265 | 3655 | 3873 | 4899 | 3983 | 2479 | 1316 | 617 | 24525 |
| 2023 | 5 | 7 | 13 | 44 | 132 | 323 | 877 | 2150 | 3627 | 3993 | 4841 | 4202 | 2609 | 1346 | 633 | 24802 |
| 2024 | 5 | 7 | 13 | 43 | 136 | 340 | 833 | 2026 | 3543 | 4230 | 4665 | 4447 | 2769 | 1381 | 653 | 25091 |
| 2025 | 6 | 7 | 13 | 41 | 140 | 361 | 797 | 1905 | 3424 | 4498 | 4463 | 4682 | 2947 | 1426 | 676 | 25386 |
| 2026 | 6 | 7 | 13 | 39 | 142 | 382 | 775 | 1796 | 3289 | 4717 | 4313 | 4871 | 3132 | 1484 | 699 | 25665 |
| 2027 | 6 | 7 | 13 | 38 | 143 | 400 | 773 | 1706 | 3152 | 4822 | 4288 | 4981 | 3318 | 1557 | 722 | 25926 |
| 2028 | 6 | 8 | 13 | 38 | 141 | 414 | 797 | 1627 | 3005 | 4807 | 4448 | 4950 | 3525 | 1653 | 747 | 26179 |
| 2029 | 7 | 8 | 14 | 38 | 137 | 428 | 843 | 1552 | 2848 | 4720 | 4743 | 4800 | 3760 | 1771 | 779 | 26448 |
| 2030 | 7 | 8 | 14 | 38 | 132 | 441 | 900 | 1494 | 2693 | 4589 | 5074 | 4624 | 3990 | 1903 | 817 | 26724 |
| 2031 | 8 | 8 | 15 | 38 | 127 | 451 | 959 | 1463 | 2555 | 4436 | 5355 | 4504 | 4184 | 2042 | 862 | 27007 |
| 2032 | 9 | 9 | 15 | 39 | 124 | 456 | 1009 | 1469 | 2444 | 4278 | 5509 | 4516 | 4310 | 2183 | 915 | 27285 |
| 2033 | 9 | 9 | 16 | 40 | 124 | 453 | 1053 | 1525 | 2348 | 4109 | 5530 | 4727 | 4315 | 2344 | 980 | 27582 |
