# Supplementary material for: Trends in incidence and mortality of laryngeal cancer in china from 2004 to 2018: Projections to 2033 and decomposition analysis
Source: PLoS One. 2025 Feb 14;20(2):e0318423. doi: 10.1371/journal.pone.0318423 (PMC11828402; doi:10.1371/journal.pone.0318423)
Supplement: S2 Table — (DOCX) [file pone.0318423.s002.docx]

**Table S2** Estimated age-specific LC cases in China female from 2019 to 2033 based on Bayesian APC prediction model

| Year | Number of age-specific LC cases | | | | | | | | | | | | | | | |
| --- | --- | --- | --- | --- | --- | --- | --- | --- | --- | --- | --- | --- | --- | --- | --- | --- |
|  | 15-19 | 20-24 | 25-29 | 30-34 | 35-39 | 40-44 | 45-49 | 50-54 | 55-59 | 60-64 | 65-69 | 70-74 | 75-79 | 80-84 | 85+ | Total |
| 2019 | 2 | 6 | 19 | 30 | 43 | 95 | 174 | 266 | 287 | 371 | 468 | 446 | 362 | 264 | 176 | 3009 |
| 2020 | 2 | 6 | 19 | 33 | 49 | 100 | 182 | 275 | 319 | 355 | 478 | 459 | 361 | 257 | 182 | 3077 |
| 2021 | 2 | 6 | 19 | 35 | 56 | 106 | 190 | 284 | 351 | 346 | 486 | 472 | 362 | 252 | 186 | 3153 |
| 2022 | 2 | 7 | 19 | 37 | 62 | 115 | 201 | 295 | 377 | 350 | 488 | 484 | 366 | 249 | 187 | 3239 |
| 2023 | 2 | 7 | 19 | 39 | 69 | 128 | 213 | 308 | 397 | 372 | 479 | 497 | 374 | 248 | 187 | 3339 |
| 2024 | 2 | 7 | 20 | 39 | 77 | 145 | 225 | 321 | 413 | 411 | 462 | 513 | 386 | 248 | 187 | 3456 |
| 2025 | 2 | 8 | 20 | 39 | 85 | 166 | 238 | 337 | 428 | 459 | 445 | 528 | 400 | 249 | 187 | 3591 |
| 2026 | 2 | 8 | 21 | 39 | 92 | 189 | 254 | 355 | 445 | 508 | 437 | 539 | 414 | 252 | 187 | 3742 |
| 2027 | 3 | 9 | 22 | 39 | 98 | 214 | 276 | 377 | 465 | 549 | 444 | 545 | 428 | 257 | 188 | 3914 |
| 2028 | 3 | 10 | 23 | 40 | 101 | 239 | 308 | 401 | 489 | 582 | 477 | 539 | 443 | 265 | 188 | 4108 |
| 2029 | 3 | 10 | 25 | 41 | 103 | 267 | 352 | 426 | 514 | 610 | 531 | 524 | 461 | 277 | 190 | 4334 |
| 2030 | 4 | 11 | 26 | 43 | 104 | 296 | 407 | 455 | 542 | 637 | 597 | 510 | 479 | 290 | 193 | 4594 |
| 2031 | 4 | 12 | 28 | 45 | 104 | 324 | 468 | 489 | 575 | 667 | 666 | 505 | 495 | 303 | 197 | 4882 |
| 2032 | 5 | 14 | 31 | 47 | 106 | 347 | 533 | 537 | 616 | 704 | 727 | 519 | 505 | 317 | 203 | 5211 |
| 2033 | 6 | 15 | 34 | 50 | 110 | 364 | 602 | 606 | 662 | 746 | 777 | 563 | 504 | 332 | 211 | 5582 |
