# Supplementary material for: Trends in incidence and mortality of laryngeal cancer in china from 2004 to 2018: Projections to 2033 and decomposition analysis
Source: PLoS One. 2025 Feb 14;20(2):e0318423. doi: 10.1371/journal.pone.0318423 (PMC11828402; doi:10.1371/journal.pone.0318423)
Supplement: S3 Table — (DOCX) [file pone.0318423.s003.docx]

**Table S3** Estimated age-specific LC deaths in China male from 2019 to 2033 based on Bayesian APC prediction model

| Year | Number of age-specific LC deaths | | | | | | | | | | | | | | | |
| --- | --- | --- | --- | --- | --- | --- | --- | --- | --- | --- | --- | --- | --- | --- | --- | --- |
|  | 15-19 | 20-24 | 25-29 | 30-34 | 35-39 | 40-44 | 45-49 | 50-54 | 55-59 | 60-64 | 65-69 | 70-74 | 75-79 | 80-84 | 85+ | Total |
| 2019 | 6 | 11 | 28 | 44 | 134 | 377 | 916 | 1275 | 1990 | 544 | 2344 | 2143 | 1741 | 1291 | 753 | 13601 |
| 2020 | 6 | 11 | 31 | 48 | 131 | 352 | 874 | 1348 | 1936 | 540 | 2522 | 2310 | 1811 | 1313 | 798 | 14035 |
| 2021 | 6 | 11 | 32 | 54 | 130 | 331 | 831 | 1400 | 1893 | 542 | 2681 | 2491 | 1901 | 1345 | 835 | 14487 |
| 2022 | 7 | 12 | 34 | 59 | 132 | 315 | 789 | 1413 | 1893 | 560 | 2802 | 2679 | 2014 | 1388 | 861 | 14962 |
| 2023 | 7 | 12 | 35 | 64 | 140 | 301 | 745 | 1388 | 1965 | 604 | 2841 | 2893 | 2158 | 1439 | 887 | 15484 |
| 2024 | 8 | 13 | 36 | 70 | 153 | 291 | 700 | 1343 | 2087 | 672 | 2804 | 3140 | 2333 | 1497 | 921 | 16073 |
| 2025 | 9 | 13 | 36 | 75 | 169 | 284 | 657 | 1287 | 2214 | 751 | 2739 | 3394 | 2531 | 1570 | 961 | 16696 |
| 2026 | 9 | 14 | 37 | 81 | 188 | 283 | 620 | 1228 | 2308 | 830 | 2692 | 3628 | 2747 | 1661 | 1004 | 17336 |
| 2027 | 10 | 15 | 38 | 85 | 208 | 290 | 592 | 1171 | 2340 | 897 | 2709 | 3812 | 2973 | 1775 | 1049 | 17971 |
| 2028 | 11 | 16 | 40 | 88 | 227 | 309 | 569 | 1112 | 2309 | 949 | 2831 | 3888 | 3234 | 1919 | 1100 | 18610 |
| 2029 | 12 | 18 | 42 | 91 | 248 | 338 | 552 | 1050 | 2246 | 994 | 3027 | 3862 | 3539 | 2095 | 1164 | 19287 |
| 2030 | 13 | 19 | 45 | 92 | 270 | 378 | 543 | 992 | 2167 | 1037 | 3233 | 3799 | 3857 | 2296 | 1240 | 19991 |
| 2031 | 15 | 21 | 48 | 94 | 290 | 423 | 545 | 942 | 2081 | 1080 | 3393 | 3765 | 4156 | 2516 | 1331 | 20712 |
| 2032 | 16 | 23 | 52 | 97 | 309 | 470 | 563 | 905 | 1998 | 1128 | 3463 | 3823 | 4401 | 2750 | 1438 | 21450 |
| 2033 | 18 | 25 | 56 | 103 | 323 | 518 | 603 | 878 | 1913 | 1179 | 3442 | 4033 | 4524 | 3025 | 1569 | 22225 |
