# Supplementary material for: Trends in incidence and mortality of laryngeal cancer in china from 2004 to 2018: Projections to 2033 and decomposition analysis
Source: PLoS One. 2025 Feb 14;20(2):e0318423. doi: 10.1371/journal.pone.0318423 (PMC11828402; doi:10.1371/journal.pone.0318423)
Supplement: S4 Table — (DOCX) [file pone.0318423.s004.docx]

**Table S4** Estimated age-specific LC deaths in China female from 2019 to 2033 based on Bayesian APC prediction model

| Year | Number of age-specific LC deaths | | | | | | | | | | | | | | | |
| --- | --- | --- | --- | --- | --- | --- | --- | --- | --- | --- | --- | --- | --- | --- | --- | --- |
|  | 15-19 | 20-24 | 25-29 | 30-34 | 35-39 | 40-44 | 45-49 | 50-54 | 55-59 | 60-64 | 65-69 | 70-74 | 75-79 | 80-84 | 85+ | Total |
| 2019 | 1 | 2 | 7 | 16 | 22 | 39 | 81 | 158 | 191 | 275 | 396 | 437 | 442 | 404 | 364 | 2835 |
| 2020 | 1 | 2 | 7 | 18 | 26 | 42 | 88 | 170 | 222 | 275 | 420 | 462 | 453 | 405 | 382 | 2973 |
| 2021 | 1 | 2 | 7 | 20 | 31 | 47 | 96 | 183 | 257 | 280 | 442 | 489 | 467 | 408 | 397 | 3127 |
| 2022 | 1 | 2 | 7 | 21 | 37 | 53 | 106 | 199 | 291 | 296 | 461 | 516 | 486 | 414 | 408 | 3298 |
| 2023 | 1 | 2 | 6 | 22 | 43 | 62 | 116 | 216 | 323 | 330 | 471 | 548 | 510 | 423 | 418 | 3491 |
| 2024 | 1 | 2 | 6 | 22 | 49 | 74 | 127 | 235 | 353 | 381 | 473 | 585 | 542 | 434 | 429 | 3713 |
| 2025 | 1 | 2 | 6 | 21 | 55 | 89 | 139 | 257 | 382 | 447 | 475 | 625 | 577 | 449 | 441 | 3966 |
| 2026 | 1 | 2 | 6 | 20 | 61 | 108 | 155 | 283 | 414 | 520 | 487 | 663 | 615 | 468 | 454 | 4257 |
| 2027 | 1 | 2 | 6 | 20 | 65 | 128 | 176 | 313 | 451 | 592 | 518 | 695 | 654 | 491 | 467 | 4579 |
| 2028 | 1 | 2 | 6 | 20 | 68 | 149 | 207 | 345 | 494 | 660 | 581 | 714 | 700 | 521 | 482 | 4950 |
| 2029 | 1 | 2 | 6 | 19 | 68 | 173 | 249 | 380 | 541 | 726 | 677 | 722 | 754 | 558 | 500 | 5376 |
| 2030 | 1 | 2 | 6 | 19 | 67 | 196 | 303 | 420 | 596 | 792 | 799 | 731 | 812 | 601 | 523 | 5868 |
| 2031 | 1 | 3 | 7 | 19 | 65 | 218 | 369 | 470 | 659 | 865 | 936 | 756 | 868 | 647 | 549 | 6432 |
| 2032 | 1 | 3 | 7 | 19 | 64 | 236 | 441 | 539 | 734 | 949 | 1075 | 811 | 919 | 695 | 581 | 7074 |
| 2033 | 2 | 3 | 7 | 19 | 63 | 246 | 521 | 639 | 817 | 1047 | 1207 | 919 | 952 | 752 | 621 | 7815 |
